# Interactive and inspectable LLM planning for human-in-the-loop EV battery disassembly

**DOI:** 10.3389/frobt.2026.1916454

**Published:** 2026-10-12

**Authors:** Marta Pedretti, Cesar Alan Contreras, Rustam Stolkin, Alireza Rastegarpanah, Roberto Pagani

**Affiliations:** 1 Department of Mechanical and Industrial Engineering, Università degli Studi di Brescia, Brescia, Italy; 2 Extreme Robotics Lab, School of Metallurgy and Materials, University of Birmingham, Birmingham, United Kingdom; 3 Department of Applied Artificial Intelligence and Robotics, School of Computer Science, Aston University, Birmingham, United Kingdom

**Keywords:** electric vehicle batteries, human-in-the-loop planning, human-robot interaction, interface design, large language models, RGB-D perception, robotic disassembly, task planning

## Abstract

**Introduction:**

Intent-driven robotic disassembly requires translating operator language and noisy RGB-D perception into grounded, precedence-consistent removal plans. This work extends a perception-grounded large language model (LLM) planning framework for two-arm electric vehicle (EV) battery disassembly and evaluates the pipeline’s effectiveness after replacing the LLM backbone.

**Methods:**

The system converts live RGB-D detections into structured scene text, rewrites coordinates into a frame-consistent LUZD representation, generates an ordered removal sequence, validates it with an LLM verifier, and applies a deterministic anti-hallucination filter that removes labels or coordinates not present in the perceived scene. The replacement LLM is first evaluated within the prior ensemble-based pipeline. The upgraded system then combines a single-checkpoint generator-verifier workflow, revised generator and verifier prompts, and an interactive, inspectable human-in-the-loop planning interface that displays intermediate outputs and final filtered plans.

**Results:**

On the same 200-scene, 600-intent benchmark used in earlier work, mean full-list correctness was 0.824 for the prior six-sample final pipeline, 0.896 when the replacement LLM was evaluated within that prior pipeline, 0.935 with the upgraded generator alone, and 0.959 with verification and filtering. The final upgraded configuration reduced the LLM-call count from seven to two. In a 12-participant within-subject pilot, mean time to accepted answer was lower on both tasks, the mean raw NASA-TLX score was lower, and all participants preferred the new interface.

**Discussion:**

These results suggest that structured scene text, clear prompts, verifier-based correction, deterministic grounding, and inspectable interfaces enhance human-in-the-loop disassembly planning.

## 1 Introduction

Robotic disassembly has become increasingly important for end-of-life products such as electric vehicle (EV) batteries, where automation can reduce costs, increase throughput, and improve worker safety (Hathaway et al., 2024). However, automating disassembly remains difficult because the scene is rarely fixed: components may appear in different configurations, perception may be incomplete or noisy, and the next feasible action often depends on precedence relations, for example, removing screws before the module they secure can be extracted. For this reason, methods based on fixed task hierarchies, handcrafted rules, or optimization-based scheduling may be difficult to adapt when the scene changes or when the operator modifies the goal during execution (Asif et al., 2024).

Recent research has investigated the use of large language models (LLMs) to infer high-level task sequences from natural-language instructions, potentially enabling flexible, intent-driven planning. However, LLMs can be brittle in robotics contexts, as they may hallucinate objects, violate strict formatting, or generate logically inconsistent sequences. To address these challenges, our pipeline evaluated intent-driven planning for EV battery disassembly using a text-based scene encoding, an ensemble of candidates sampled from a single LLM checkpoint, an LLM verifier, and a final deterministic filter (Erdogan et al., 2026). That study reported accuracy improvements from a single-model baseline to an ensemble plus verification pipeline, but this approach required additional model calls and parallel candidate generation as the ensemble size increased.

The planning task addressed in this work is defined as follows. At each time step t , the system receives (i) a structured scene representation $\sigma_{t}$ derived from perception, containing object labels and their spatial attributes, and (ii) a user instruction $U_{t}$ expressed in natural language. The objective is to generate an ordered action list $A_{t}$ , where each element corresponds to a valid object removal operation.

The generated plan must satisfy three constraints. First, it must be complete, including all required objects and any blocking prerequisites. Second, it must be correctly ordered, ensuring that precedence constraints are respected and blocking objects are removed before the objects they obstruct. Third, it must be grounded in the perceived scene, such that every referenced object corresponds exactly to an element present in the structured scene representation $\sigma_{t}$ . This formulation enables the evaluation of planning as a constrained sequence-generation problem.

This study reports two separate evaluations: offline benchmark results, which measure planning performance on the clean dataset, and live deployment results, which assess the human-in-the-loop interface during interactive use. While this work builds directly on the planning pipeline introduced in (Erdogan et al., 2026), the present study transforms the system into an inspectable human-in-the-loop planning tool. In contrast to the prior ensemble-based approach, this study adopts a single instruction-following model, revised generator and verifier prompts, a stricter deterministic grounding filter, and a fully integrated user interface. The main contributions are:

First, a new web interface is presented that (i) streams 3D perception and detections, (ii) displays the exact text sent to each LLM stage, and (iii) provides stage-by-stage inspection of raw, cleaned, and verified plans. This approach reduces operator friction and accelerates debugging compared with previous workflows. Second, the study first evaluates the replacement LLM within the prior pipeline and then evaluates an upgraded configuration with revised generator and verifier prompts, instance identifiers, and deterministic grounding (OpenAI, 2023; Grattafiori, 2024; Yang, 2024; Gemini Team, 2024). Third, system reliability in full-list evaluations is improved by updating the generator and verifier prompts to emphasize clear formatting (Zhou et al., 2023; Jiang et al., 2024; Dussolle et al., 2025), and by employing a deterministic anti-hallucination filter^1^ (Manakul et al., 2023; Huang et al., 2025) to ensure the plan matches the scene.

## 2 Related work

### 2.1 LLMs for embodied planning and manipulation

Embodied and robotics-oriented LLM systems typically address the gap between language and physical state via grounding mechanisms: embedding state into the LLM input (structured text, images, or state vectors), and constraining outputs to actionable representations. PaLM-E demonstrated multimodal embodied reasoning by interleaving continuous state and visual tokens into a language model (Driess et al., 2023). RT-2 showed that vision-language-action models can transfer web knowledge to robot control (Zitkovich et al., 2023). More recent systems combine LLM task reasoning with geometric planning; for instance, 3D-LOTUS++ integrates 3D-LOTUS’s motion planning capabilities with LLM task planning and VLM grounding (Garcia et al., 2025). ManipLLM targets object-centric manipulation with test-time adaptation and commonsense reasoning chains (Li et al., 2024). Our work focuses on disassembly and emphasizes conventional verification and data consistency, rather than tighter integration between LLM planning and downstream task-and-motion execution (Garcia et al., 2025; Wang et al., 2024).

### 2.2 Instruction following and constrained outputs

In LLM planning pipelines, the model must often satisfy strict instructions on format, output length, and task constraints. Benchmarks such as IFEval, FollowBench, and M-IFEval formalize verifiable constraints (for example, “output only a list”) and demonstrate that instruction-following varies substantially across models (Zhou et al., 2023; Jiang et al., 2024; Dussolle et al., 2025). In response, this extension strengthens the output contract and aligns generator and verifier prompts around a shared formal grammar.

### 2.3 Hallucination detection and mitigation

In robotic settings, an LLM may refer to objects that are not present in the input state, which can lead to invalid or unsafe actions. Previous surveys describe hallucination as a multi-cause problem and discuss mitigation strategies such as constrained decoding, retrieval grounding, and *post hoc* verification (Huang et al., 2025). SelfCheckGPT introduces a sampling-based consistency check for black-box models (Manakul et al., 2023). In robotics, post-processing filters that enforce input-output consistency are particularly attractive because they are cost-effective, deterministic, and can be integrated as a final safety gate. The anti-hallucination filter in this work performs exact token-level grounding against the perceived scene payload.

### 2.4 EV-battery disassembly context

Robotic disassembly and recycling are actively studied due to safety, cost, and throughput requirements. Recent surveys and technoeconomic analyses show that EV-battery disassembly offers clear opportunities, but also practical constraints, including uncertainty, non-standardized designs, and frequent human-in-the-loop requirements (Asif et al., 2024; Hathaway et al., 2024). Disassembly planning is also related to multi-robot scheduling and safety-aware coordination under uncertainty (Fatemi-Anaraki et al., 2023; Wang and Chen, 2024; Pan et al., 2024), with semantic knowledge supporting planning beyond purely geometric heuristics (Bernardo et al., 2023). Intent-driven LLM planning has been investigated for EV-battery disassembly (Erdogan et al., 2026), and the present study retains the prior generator-verifier-filter structure while replacing the LLM backbone and revising the prompts and operator interface.

## 3 System overview

Figure 1 shows the overall structure of the proposed pipeline. The process begins with a synchronized RGB-D frame, where a 2D object detector (YOLOv11) is applied to the RGB image to obtain class labels and 2D bounding boxes (Ultralytics, 2024). For each detection, the bounding box serves as a region of interest in the depth image. Valid depth pixels within the box are selected, median image coordinates and depth values are computed, and the resulting representative pixel is back-projected through the camera intrinsics to estimate a metric 3D position (x,y,z) in the camera frame.

**FIGURE 1:**
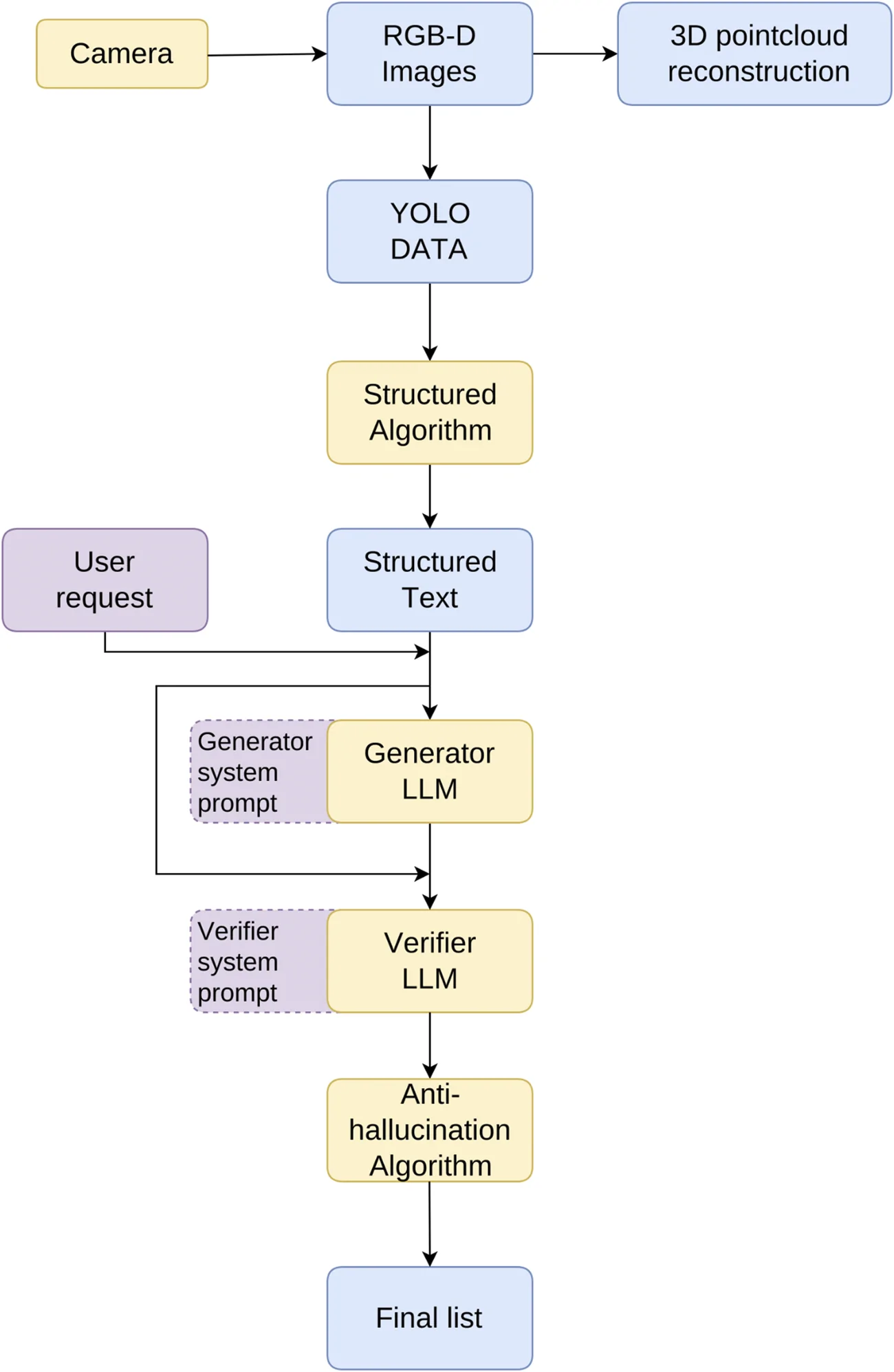
End-to-end pipeline: the user request and perception-grounded structured text (RGB-D/3D/YOLO) are processed by generator/verifier LLMs and an anti-hallucination module to produce the final disassembly list.

The resulting detections are first serialized as plain scene text, with each item represented by a line containing its label and centroid coordinates. These entries are then rewritten into a structured representation, where coordinates are normalized to a *LUZD* form and strict formatting constraints are enforced.^2^

This structured scene text, combined with the user request, serves as input to the generator LLM, which operates under instructions provided via a fixed system prompt. The generator LLM first produces a list-only ordered removal plan. Subsequently, a verifier LLM re-evaluates and corrects the plan according to explicit validation rules. The verifier is conditioned on both the user request and the scene description, with instructions delivered through a fixed system prompt that includes the generator prompt. Finally, a deterministic anti-hallucination filter retains only those output entries whose object references can be exactly matched to items in the source scene text $\sigma_{t}$ .

The proposed system operates under three primary assumptions. First, the perception module provides sufficiently accurate object detections and approximate spatial localization. Second, the structured scene representation is assumed to contain all objects that may affect the removal plan, including potential blockers and prerequisite objects. Third, the defined matching rules are assumed to map the user instruction to a valid target in the scene. Violations of these assumptions, such as missing detections, inaccurate localization, or ambiguous instructions, may result in incomplete, empty, or invalid plans.

The pipeline is evaluated in two ways. The first is an *offline benchmark* using previously cleaned, structured scene text from the established dataset (Erdogan et al., 2026). The second evaluation mode is *live deployment*, which begins with RGB-D cameras mounted on the robot. YOLOv11 detection is performed, followed by application of a depth-fusion pipeline. Per-detection 3D positions are estimated from the median depth over valid pixels, detections are serialized into scene text, coordinates are rewritten into *LUZD* form, and the generator-verifier-deterministic-filter pipeline planning chain is executed. In this study, “live deployment” denotes sensing and perception from live RGB-D data, planning through the deployed model endpoint, interface operation, plan inspection, and operator acceptance under controlled laboratory conditions.

### 3.1 Baseline pipeline

The baseline framework (Erdogan et al., 2026) employs a perception-grounded planning pipeline. Synchronized RGB–D streams are acquired by wrist-mounted Intel RealSense cameras on two UR10e arms. The sensor data is segmented using YOLOv8 to obtain object detections, which are rendered into an unstructured scene string and then structured and serialized into a group-based scene text. Each Cartesian coordinate (x,y,z) in the structured text is rewritten into a form *LUZD* that aligns with the selected spatial convention. The transformed scene text is combined with the operator’s natural-language instruction in a user message. A fixed generator system prompt specifies a strict list-only output contract and a target-resolution procedure for generating an ordered removal plan. Multiple candidate plans are produced by sampling the same model with different random seeds. A verifier pass then invokes the model under a dedicated verifier system prompt. The verifier receives the original context (generator prompt and user message) along with the set of candidate lists, evaluates them against explicit formatting, presence, and ordering criteria, and returns the first fully compliant list or an empty output if none satisfy all constraints. The verified list is parsed to extract object labels and coordinates, which are mapped back to the original (x,y,z) frame. Then, a deterministic filter retains only entries whose text matches verbatim an item in the original structured scene text, thereby removing hallucinated objects.

Table 1 presents the baseline results as reported in (Erdogan et al., 2026). The *Full* column represents the full-list correctness metric, which is also employed in the present study. This metric is computed using the same dataset, ground-truth sequences, scoring, and rules. Consequently, the baseline values in Table 1 are directly comparable to the improved pipeline results discussed later. Under this metric, a prediction is considered correct only if the full list matches the reference solution in both content and order. The *Next* column reports next-object correctness, measuring whether the first predicted removal action is correct. Although this metric was included in the baseline study, it is not an evaluation criterion in the current work. *Single* refers to one LLM generator, *Ensemble* refers to multiple candidate lists sampled from the same LLM model, and *Final* refers to the output of the ensemble after verifier LLM and deterministic filtering.

**TABLE 1:** Baseline pipeline results (full-list and next-object correctness) from the same executions in (Erdogan et al., 2026).

LLMs	Stage	Full	Next	Avg time (s)
1	Single	0.761	0.866	5.571
3	Ensemble	0.787	0.863	21.934
3	Final	0.796	0.871	22.153
6	Ensemble	0.816	0.888	32.504
6	Final	0.824	0.894	32.744

### 3.2 Retained components from the baseline pipeline

Several components from the baseline pipeline are incorporated without modification in the proposed approach. The following remain unchanged:

#### 3.2.1 P1: structured inputs

Instead of processing raw detector outputs, the LLM receives a compact scene description. Specifically, the unstructured YOLO data, which consists of per-detection records with fields such as <label>, <confidence>, <bbox>, and metric coordinates, is rewritten by a post-processing algorithm into structured, group-based text. Additionally, to reduce input variability, a parent-child grouping (tower rule) is inferred from bounding-box nesting: a detection whose image-space center lies within another bounding box is assigned to that bounding box as its child. This yields group-wise descriptions in which a root object is listed together with its associated child items using a fixed template (e.g., *“with* *screw* *,* *screw* *on top of it”*).

A minor modification is introduced compared to the previous pipeline. Rather than referring to objects solely by their class label, each detected object is assigned a specific instance identifier. This means that each object is represented by <label_instance#> rather than only by <label>, so that multiple detections of the same class can be selected and referred to separately.

#### 3.2.2 P2: coordinate rewriting

All Cartesian triples (x,y,z) present in the structured scene text are rewritten into a four-parameter form as defined in Equation 1.

$$\left[L,U,Z,d\right]=\left[-z,\;x,\;y,\;\sqrt{x^{2}+y^{2}+z^{2}}\right],$$ (1)

This transformation produces a rewritten scene description, as expressed in Equation 2:

$$D_{t}=T\left(\sigma_{t}\right).$$ (2)

The resulting representation follows the selected spatial convention, while non-coordinate tokens are left unchanged. As in the baseline, coordinate rewriting is used as a normalization step to reduce ambiguity in spatial references (Erdogan et al., 2026).

#### 3.2.3 P3: strict output requirements

The generator and verifier prompts allow only one output format and exclude additional commentary. This follows the same principle used in instruction-following evaluations (Zhou et al., 2023; Jiang et al., 2024), where explicitly checkable constraints are generally easier to enforce than open-ended requirements.

Prompt constraint: list-only output.

STRICT RESTRICTION: Your response must contain ONLY the labels and coordinates of the parts to be removed.
- DO NOT provide explanations.
- DO NOT provide headings or section titles.
- DO NOT provide numbering.
ABSOLUTE OUTPUT RULE
Emit only the lines:
<Label_instance#> [L, U, Z, d]
<Label_instance#> [L, U, Z, d]
.
No bullets, no blank lines, no extra text.

Example model output.

Model output:
screw_1 [-0.750, 0.240, 0.020, 0.788]
screw_2 [-0.760, 0.350, 0.015, 0.837]
LeafCell_1 [-0.780, 0.310, -0.040, 0.840]

#### 3.2.4 P4: safety grounding

A deterministic check filters any output line whose label and coordinate tuple do not match the structured input. This process ensures that, even in cases of model hallucination, the final output remains grounded in the real scene content.

## 4 Extension: backbone-replaceable planning and improved reliability

This section outlines the extension of the baseline to a more reliable planning stack.

### 4.1 Perception grounding and fusion

Prior to planning, depth data is temporally stabilized and fused to improve robustness in live RGB-D capture. For a window of W synchronized frames, raw depth at pixel (u,v) is aggregated as shown in Equation 3:

$$z_{t}^{\text{raw}}\left(u,v\right)={median}_{k=1,\ldots ,W}\;z_{k}\left(u,v\right),$$ (3)

and its temporal dispersion is estimated via the median absolute deviation (MAD), as defined in Equation 4:

$${MAD}_{t}^{\text{raw}}\left(u,v\right)={median}_{k}\left|z_{k}\left(u,v\right)-z_{t}^{\text{raw}}\left(u,v\right)\right|.$$ (4)

DepthAnything-v3 (Lin et al., 2026) is applied to the same window and averaged to produce a denoised relative-depth prediction $z_{t}^{\text{da}3}\left(u,v\right)$ and associated confidence $c_{t}^{\text{da}3}$ . To recover metric scale, the DA3 output is aligned to the raw depth using the global scale-shift model in Equation 5:

$${\hat{z}}_{t}\left(u,v\right)=a\;z_{t}^{\text{da}3}\left(u,v\right)+b,$$ (5)

where (a,b) are estimated via scale-shift regression over sampled valid pixels. Valid pixels are defined as those with finite raw depth within the accepted depth range. The pixel sample fraction is denoted by $f_{\text{sample}}$ , and MAD-based inlier rejection is controlled by $k_{\text{MAD}}$ .

The aligned prediction ${\hat{z}}_{t}$ is then corrected using a weighted residual field derived from the discrepancy defined in Equation 6:

$$r_{t}\left(u,v\right)=z_{t}^{\text{raw}}\left(u,v\right)-{\hat{z}}_{t}\left(u,v\right),$$ (6)

yielding the fused depth map in Equation 7:

$$z_{t}^{\text{fused}}\left(u,v\right)={\hat{z}}_{t}\left(u,v\right)+clip\;\left(\Delta_{t}\left(u,v\right),-\delta ,\delta \right),$$ (7)

where $\Delta_{t}\left(u,v\right)$ represents a locally smoothed, reliability-weighted estimate of $r_{t}\left(u,v\right)$ , which upweights pixels with stable raw depth (low MAD) and high DA3 confidence. Only pixels with valid raw-depth support are used for correction and subsequent processing, ensuring that new 3D points are not introduced where raw depth is absent. The fused depth is utilized to compute metric centroids, and is also employed for live 3D reconstruction displayed in the UI. Figure 2 provides an overview of the depth-fusion pipeline.

**FIGURE 2:**
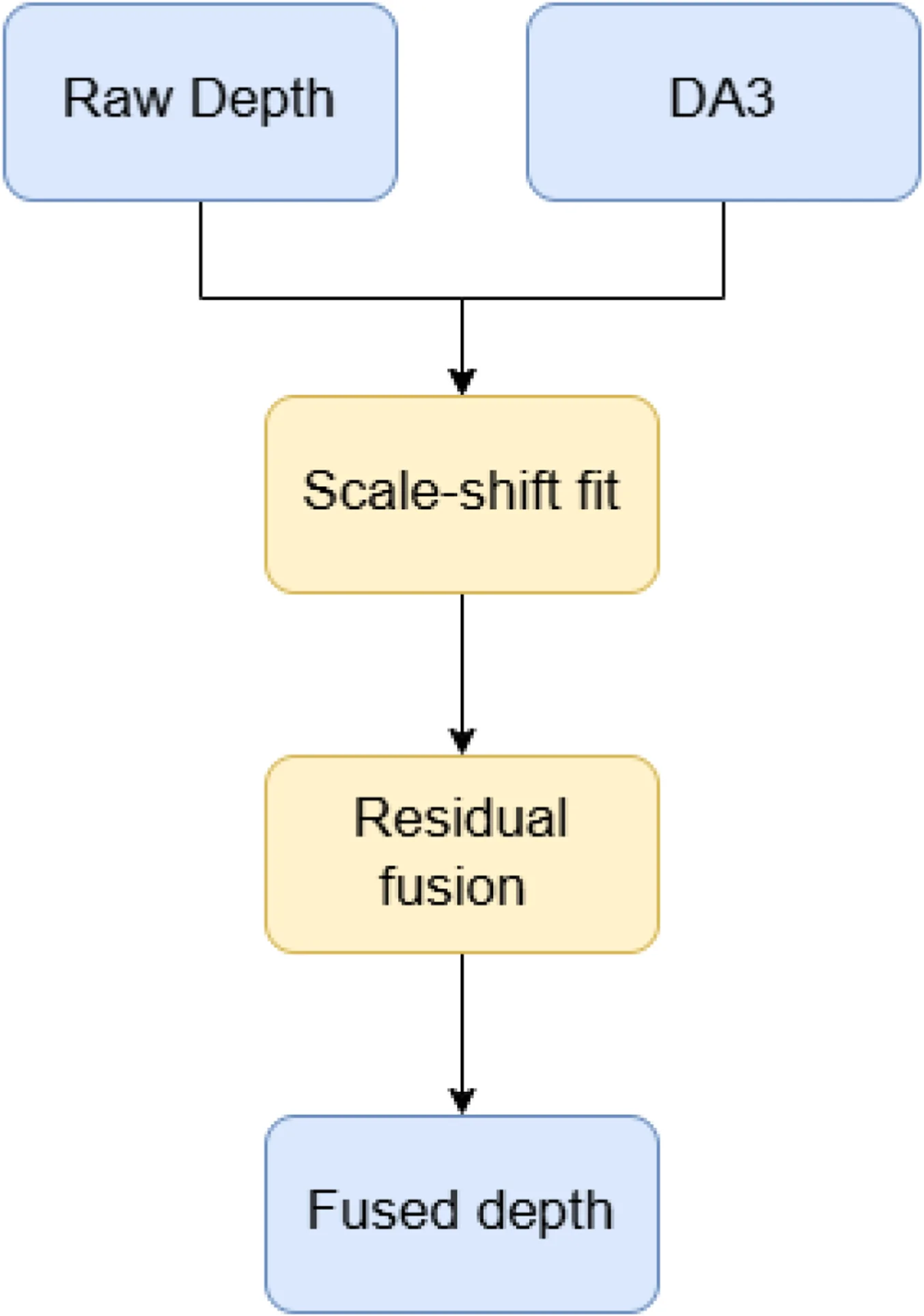
Diagram of the depth-fusion pipeline used to generate stable metric 3D information and the final 3D scene view.

The offline benchmark bypasses this module because the planner receives cleaned structured scene text. During live operation, fused depth supports centroid estimation, scene-text generation, point-cloud visualization, and operator inspection. Additional implementation safeguards are incorporated to enhance the stability of the fused depth estimate and mitigate the effects of local noise, outliers, and depth discontinuities. Nevertheless, the planning stage remains contingent on the quality of the perception output. Perception errors, including missed detections or inaccurate depth estimates, directly impact the structured scene representation and may propagate to the planning stage. Specifically, missing objects cannot be recovered by the planner, and incorrect spatial relationships may result in invalid ordering decisions.

### 4.2 Selection and deployment of the LLM

In the baseline system, inference was performed locally on a dedicated workstation equipped with an Intel i9 CPU, an NVIDIA RTX 5090 GPU, and 32 GB of RAM. This workstation hosted a 4-bit quantised Qwen3-32B model through Ollama. Perception and robot-control processes operated on a separate robot machine with an Intel i7 CPU.

The upgraded pipeline replaces the locally hosted baseline model with moonshotai/Kimi-K2-Instruct-0905, served through the Featherless OpenAI-compatible API. This larger instruction-following Mixture-of-Experts model was selected during iterative development and then fixed for the reported evaluations. The model is first evaluated within the prior pipeline and then used in three upgraded configurations: Gen, Gen + Ver, and Gen + Ver + alg.-filter. Because model and prompt selection used the benchmark during system development, the offline results are reported as regression performance.

Generator and verifier requests used temperature=0.2, top_p=0.4, top_k=-1, min_p=0.06, repetition_penalty=1.04, max_tokens=4096, and num_ctx=32768. Generator and verifier seeds were 21 and 2025, respectively, and streaming was enabled for interface display. Supplementary Appendix Table A7 reports the request configuration.

The evaluation first applies the replacement LLM to the prior multi-sample ensemble pipeline. The upgraded configurations then replace ensemble sampling with one generator call and, when verification is enabled, one verifier call. The structured scene representation, verifier stage, and deterministic grounding filter remain modular pipeline components. Supplementary Appendix Table A7 reports the provider, model identifier, decoding parameters, context length, and seeds used in the upgraded pipeline.

### 4.3 Revised generator system instructions

The generator system prompt was revised while preserving the same strict list-only output format and ordering rules. Compared with the baseline version, the new prompt follows a clearer step-by-step procedure: it first parses the user intent, then resolves the target object, identifies any required prerequisite removals, and finally fixes the output order before generation begins. This was done to make the generation process more structured and reproducible.

The resolution stage was made more conservative. Exact label matching remains the preferred approach, but a limited fallback matching rule is applied for underspecified object names. Permitted transformations include case-insensitive matching and underscore (_) removal. A target is accepted only if these rules lead to a single unambiguous match in the current scene. If multiple candidates remain possible, the generator does not attempt to guess and instead returns the output NONE []. During evaluation, NONE [] is considered incorrect unless the ground-truth solution is also empty. The prompt also addresses generic requests that do not specify a unique instance or locator. In these cases, wildcard-style matching is permitted only if it results in a single unambiguous object class in the current scene. Otherwise, the request is considered ambiguous and is mapped to NONE [].

The obstruction logic is extended through an extended tower rule. In addition to standard vertical stacking constraints, an item is considered a blocker if it is within a fixed lateral distance threshold of the target, as measured in the planner coordinate frame. This proximity check applies only to planar coordinates, while vertical precedence continues to be managed by the standard tower rule. Finally, the prompt includes an explicit self-check before emission, requiring the final ordered list to be fixed before output starts. The empty-action case is always represented as NONE [].

### 4.4 Revised verifier system prompt

The verifier prompt transitioned from a candidate-selection role to a requirements/structure validation and minimal-correction role. In this framework, the generator proposes a candidate plan based on structured input and user intent, while the verifier enforces compliance with the specification by correcting structural, formatting, and ordering errors.

In the baseline configuration, the verifier receives the structured GROUP BLOCK, the user request, and N≥1 generator candidates (Response 1.N). It recomputes the canonical solution by re-running the same algorithm as the generator, validates each candidate against explicit rules (syntax, verbatim presence in the group block, exact list equality, and duplicate constraints), and returns the first passing candidate unchanged. If no candidate passes, the verifier returns an empty output.

In the updated configuration, the verifier receives the GENERATOR PROMPT (SPEC) as a binding contract, the user request (EXTRA PROMPT), and a single candidate list (PREVIOUS RESPONSE). The verification contract enforces a minimal-change policy: if the candidate list fully complies with the generator specification and satisfies the user intent, it must be emitted identically; otherwise, the verifier produces a corrected list by applying the minimal set of edits required. The updated prompt also mandates strict non-expansiveness on the list.

Failure handling differs between the two versions. The baseline verifier returns an empty output when no candidate passes validation. In contrast, the updated verifier restricts outputs to either (i) a list in the prescribed format or (ii) exactly one allowed error string defined by the generator specification.

### 4.5 Anti-hallucination filter

The post-check concept is adopted from (Erdogan et al., 2026), but is refined here to require exact grammatical grounding with respect to the scene input. Each verifier line is parsed as $\left(\ell^{\left(n\right)},q^{\left(n\right)}\right)$ with $q^{\left(n\right)}=\left(L,U,Z,d\right)$ . The coordinates are back-mapped to XYZ as (x,y,z)=(U,Z,−L) and matched against the structured scene text $\sigma_{t}$ for the current scene at three-decimal precision using the acceptance indicator in Equation 8:

$$\chi_{t}^{\left(n\right)}=\chi \left(\ell^{\left(n\right)},q^{\left(n\right)};\sigma_{t}\right)\in \left\{0,1\right\}.$$ (8)

where $\chi_{t}^{\left(n\right)}=1$ only if the line satisfies the strict regex format and its label and coordinates are present in the current scene text $\sigma_{t}$ , i.e., the structured source text built from the objects present in that scene. The final accepted list is defined in Equation 9:

$$A_{t}=uniq\left\{\left(\ell^{\left(n\right)},q^{\left(n\right)}\right)|\chi_{t}^{\left(n\right)}=1\right\}.$$ (9)

Here, uniq(⋅) denotes an order-preserving deduplication step: if the same accepted entry appears multiple times, only its first occurrence is retained in $A_{t}$ .

A deterministic grounding filter is adopted to ensure predictable and verifiable behavior. The deterministic approach applies a fixed rule: an output line is accepted only if its label and coordinates can be matched to the current scene representation. This deterministic gate verifies scene membership but not plan completeness, precedence correctness, or executability. Outputs cannot introduce unseen objects, even when LLM reasoning is inconsistent. The filter does not ensure that all required prerequisites remain in the final list. In the offline benchmark, omission of any required object makes the full-list prediction incorrect. During live use, the filtered result remains an operator-inspected proposal.

The overall design favors simple checks over more complex learned components. In the perception stage, temporal median filtering, MAD-based rejection, and scale-shift depth alignment are used because their behavior is easy to inspect and debug. In the planning stage, strict formatting rules, exact label grounding, and deterministic coordinate matching are used because uncertain outputs should be rejected before they can be treated as robot commands. This choice reduces flexibility, since valid outputs may be rejected if their format or coordinates differ from the expected representation, but it provides a clearer safety boundary for human-supervised disassembly planning.

## 5 Web interface design

A common limitation of many LLM-planning demonstrations is that operators cannot easily see how a plan was produced, whether it is grounded, or which stage caused an error. To address this, a web-based interface has been developed that organizes the workflow around three practical steps: updating the scene, reviewing the detections, and inspecting the generated plan. In one workspace, operators can run a scan, update detections, remove spurious objects, write or build an instruction, and inspect the structured input, generator output, verifier output, and final filtered list. The aim is to make the planner easier to supervise, not to hide the planning process from the operator during live system use and review.

In the baseline UI (Figure 3), operator intent is mainly entered as free text (“Extra prompt”), so results can depend on wording and vary across users or runs. In the extended UI (Figure 4), the first design change is object buttons and scene curation: detected objects are shown as clickable items with class names and numeric IDs, so the instruction can be built by selecting objects instead of retyping names. This reduces typing errors, gives the operator a direct link between the request and the perceived scene, and is consistent with HCI work on direct manipulation and prompt-support tools (Masson et al., 2024; MacNeil et al., 2023; Arawjo et al., 2024). The same interface allows operators to remove or hide unwanted detections before planning. In this way, false or low-quality perception outputs become correctable intermediate results rather than errors that silently affect the final decision (Endsley, 2023; Wilchek et al., 2023).

**FIGURE 3:**
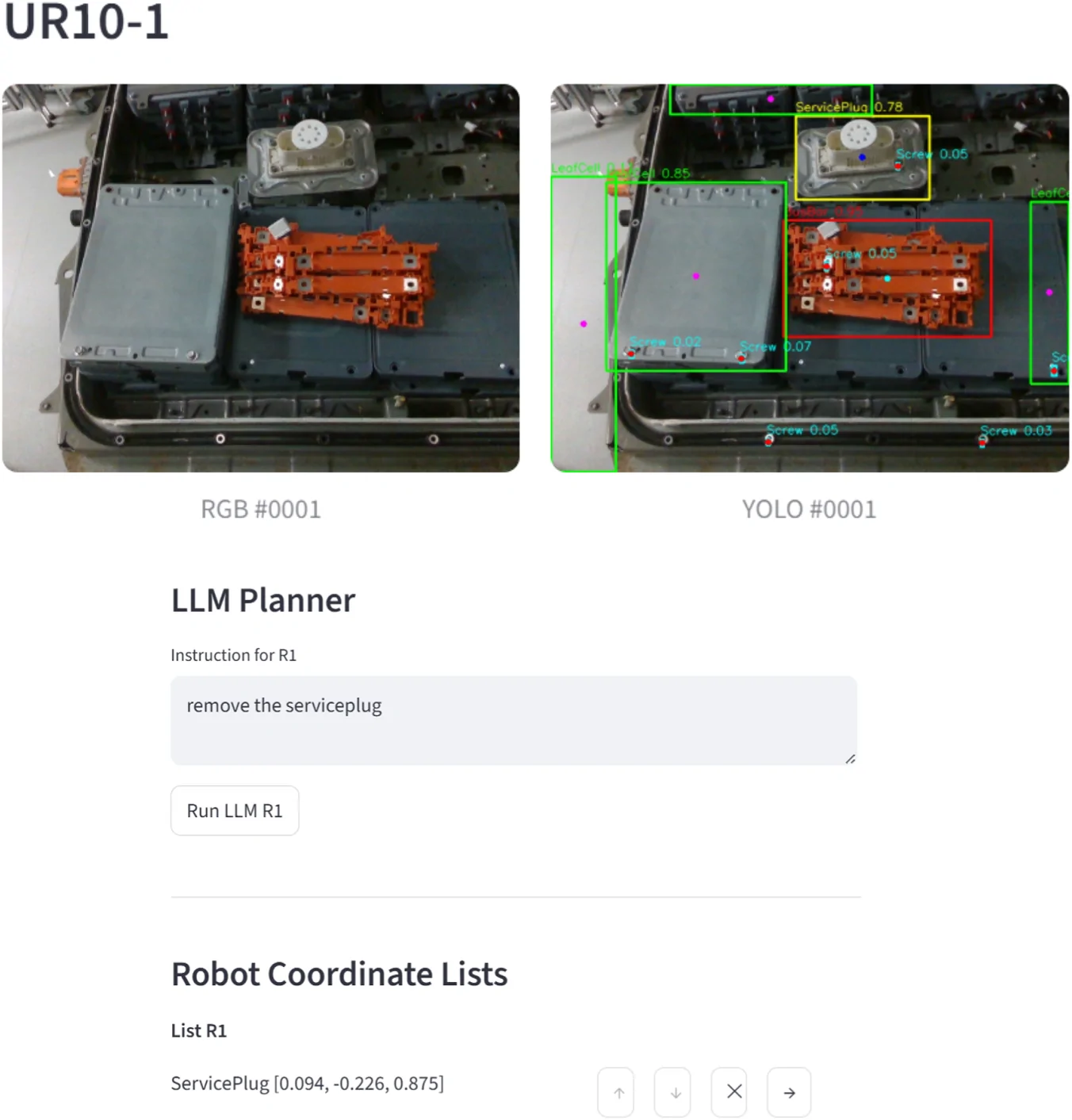
Baseline UI (two vertical snapshots). The detections view and the instruction authoring stage are located in different sections of the page, so the operator must scroll to move from perception results to planning and output inspection.

**FIGURE 4:**
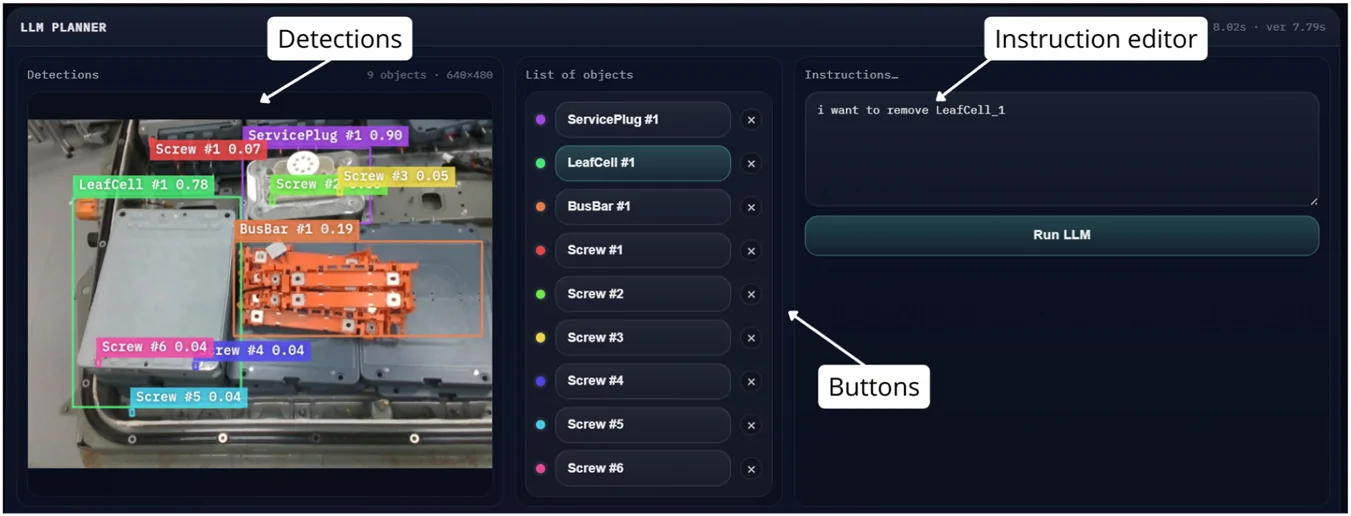
Extended UI. Detections, object-level controls (including removal/curation), and the instruction editor are co-visible within a single viewport, supporting a tighter perception-to-instruction loop without scrolling.

The baseline interface places detections, prompt entry, and intermediate text in a vertically stacked layout, often forcing users to scroll between non-adjacent areas. In the extended UI, co-located panels and visual matching keep the 2D detection preview, object list, and instruction editor visible in one viewport and ordered to match the workflow: perception, selection, instruction, and output inspection. This reduces visual navigation and split attention, which can help situational awareness and reduce cognitive load when users must combine several information sources quickly (Cheng et al., 2024; Zhang et al., 2024). The UI also uses a dark, low-glare background. Each object bounding box is linked to the corresponding control through both color and a numeric ID. Since object identity is also encoded through labels and numbers, the operator does not need to rely on color alone; this is useful both for repeated object-to-control matching and for users with color vision deficiency (Jamil and Denes, 2024; Geddes et al., 2025). Consistent cues across icons, labels, and controls can also reduce visual search effort (Zhang et al., 2022), while dark interfaces may reduce subjective strain during longer use (Pathari et al., 2024; Sengsoon and Intaruk, 2025). These choices are simple, but they make repeated object checking less dependent on memory and manual comparison.

To support spatial understanding, the extended UI adds 3D and depth inspection through a point-cloud view and side-by-side raw and fused depth views (Figure 5a). This helps operators verify that the scene geometry is reasonable before trusting the generated list, especially when depth noise or occlusion can alter the apparent object positions. It also supports judgement of relative position, occlusion, and reachability (Mazeas and Namoano, 2025; Schwenk and Smith, 2025). Showing raw and fused outputs side by side, with the option to rerun fusion, lets operators see where depth enhancement improves geometric consistency and where it may introduce artifacts. The fusion stage uses a “Depth Anything” monocular depth model to densify or refine depth estimates in difficult regions (Lin et al., 2026).

**FIGURE 5:**
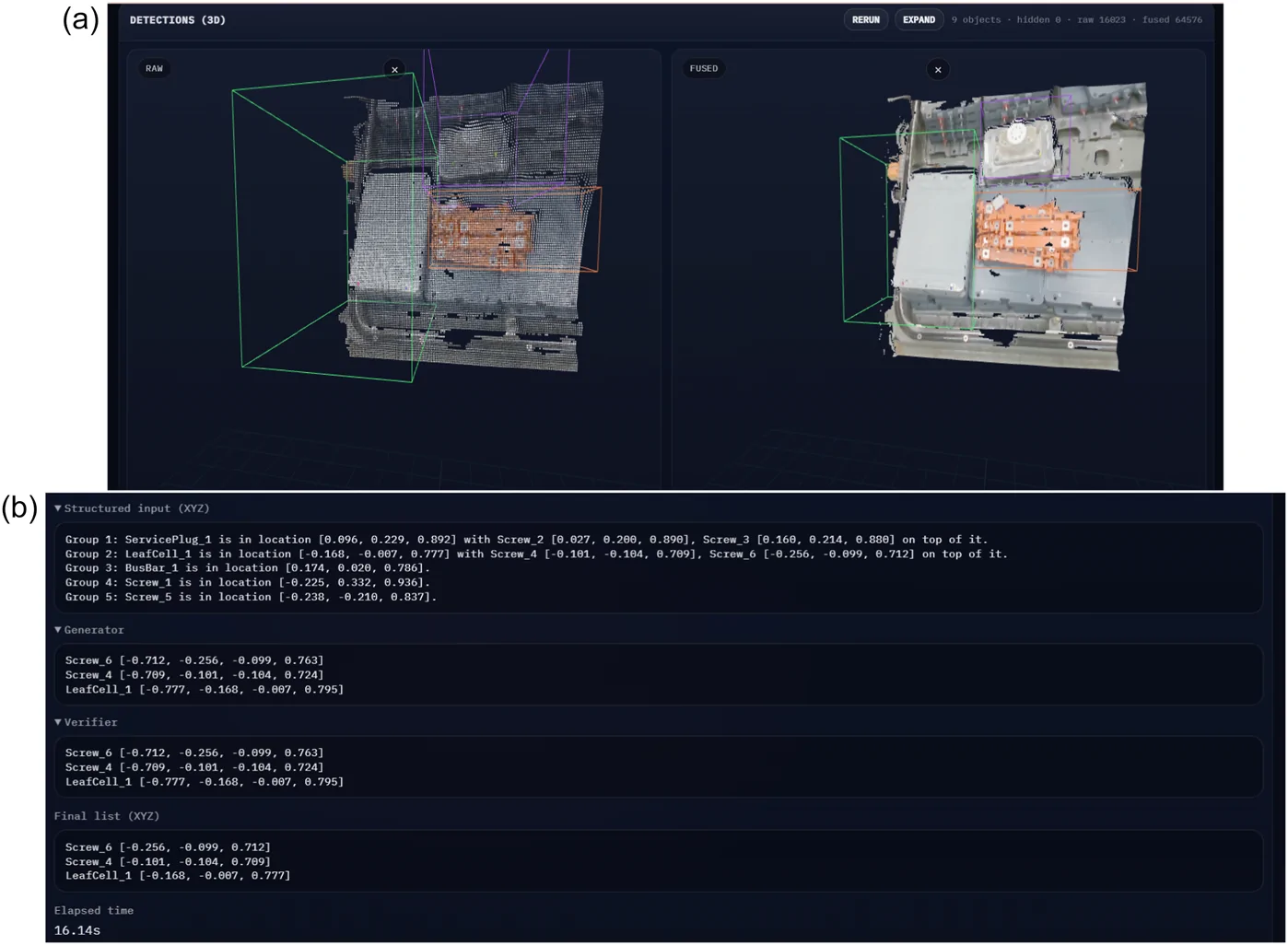
Extended UI diagnostic views. **(a)** 3D point-cloud reconstruction with raw vs. fused depth for spatial verification. **(b)** Inspectable generation trace showing structured input and intermediate outputs (generator, verifier) leading to the final list.

The baseline UI gives limited visibility into how the final list is produced. In the extended UI, the inspectable generation trace shows the structured input, generator output, verifier output, discarded items, and latency breakdown (Figure 5b). By exposing the intermediate trace, the interface lets the operator localize a failure to a specific stage, such as perception formatting, generation, verification, or final filtering. The operator can then decide whether the current list can be accepted, or whether the instruction should be edited, the detections curated, or the planner run again. This design choice is aligned with work on AI transparency, where visible intermediate states are used to support user understanding, trust calibration, and error recovery in interactive AI systems (Liao and Wortman Vaughan, 2024; Jantzen et al., 2024; He et al., 2025).

## 6 Evaluation

We evaluate the planning system in two ways: (i) *planning accuracy* on an offline cleaned benchmark, used to isolate changes in the LLM planning module, and (ii) *operator usability* in live deployment, used to compare the human-in-the-loop workflow between the old GUI and the new GUI.

### 6.1 Offline accuracy evaluation on the cleaned benchmark

#### 6.1.1 Metrics

Prior work reported two metrics on the cleaned benchmark: full-list correctness and next-object correctness (Erdogan et al., 2026). Full-list correctness checks whether the whole ordered plan matches the ground truth, while next-object correctness checks only whether the first removal action is correct. In this work, we use full-list correctness as the main metric. A prediction is correct only if it returns the complete ordered removal list, including (i) all required objects and blocking prerequisites, (ii) the correct order, so that blockers are removed before the objects they block, and (iii) only scene-grounded label + coordinate pairs that correspond to detected objects.

#### 6.1.2 Experimental protocol

The offline evaluation contains two comparisons. First, the replacement backbone is evaluated within the prior pipeline. Second, the upgraded pipeline is evaluated as Gen, Gen + Ver, and Gen + Ver + alg.-filter. All conditions use the same 600 scene-query pairs, user intents, ground-truth lists, full-list correctness definition, admissibility rules, and scoring procedure used in Erdogan et al. (2026).

These 600 scene-query pairs are reused from the prior benchmark to enable a direct regression comparison with the baseline pipeline. No model weights were trained or fine-tuned on these data. The results quantify the performance of the upgraded pipeline on the benchmark established in the earlier study.

The replacement backbone is first evaluated within the prior six-sample final pipeline. The upgraded pipeline is then tested in three configurations: (i) Gen, using only the generator; (ii) Gen + Ver, using the generator followed by the verifier; and (iii) Gen + Ver + alg.-filter, which also applies the deterministic anti-hallucination grounding filter. Each configuration is evaluated in three independent batches over the same 600 scene-query pairs, where each pair contains one scene and one user instruction. Since the inputs and scorer do not change within this upgraded-stack comparison, variation across batches mainly reflects generation variability, while differences between configurations reflect the incremental addition of the verifier and grounding filter. The prior pipeline using the replacement LLM provides a reference for assessing the remaining pipeline changes as a group rather than as individually isolated effects. All offline values are reported descriptively as means over three repeated stochastic batches.

#### 6.1.3 Component study: generator, verifier, and grounding filter

Table 2 shows how full-list correctness changes as verifier and deterministic grounding stages are added within the upgraded pipeline. The generator-only configuration reaches 93.53% mean full-list correctness, showing that the upgraded generator, revised prompt structure, and structured scene text already support reliable list generation. Adding the verifier (Gen + Ver) raises correctness to 94.70%. This is consistent with the verifier correcting format drift and ordering errors, such as missing prerequisite removals or incorrect prerequisite order. Adding the anti-hallucination grounding filter (Gen + Ver + alg.-filter) gives the best result, 95.90%, corresponding to a +2.37pp increase over Gen-only and a +1.20pp increase over Gen + Ver.

**TABLE 2:** Mean full-list correctness on the offline benchmark for the prior final pipeline with the replacement LLM and for the upgraded-stack component study. alg.-filter denotes the deterministic anti-hallucination grounding filter applied to the verifier output.

Setting	Mean (%)
Prior final pipeline, replacement LLM	89.60
Upgraded gen only	93.53
Upgraded gen + ver	94.70
Upgraded gen + ver + alg.-filter	95.90

Within the prior pipeline, mean full-list correctness was 82.40% with the original LLM and 89.60% with the replacement LLM, a difference of 7.20 percentage points. With the replacement LLM fixed, the final upgraded configuration reached 95.90%, 6.30 points above the prior-pipeline result. This second difference combines the revised generator prompt, instance identifiers, updated verifier behavior, removal of ensemble sampling, and deterministic grounding. Within the upgraded stack, adding the verifier increased mean correctness by 1.17 points, and adding the grounding filter provided a further 1.20-point increase.

#### 6.1.4 LLM-call count comparison

Table 3 compares the number of LLM calls required by the prior 6-sample ensemble pipeline and by the upgraded variants. The prior 6-sample “Final” pipeline uses six generator calls to produce candidate plans and one verifier call to select the final answer, for a total of seven LLM calls. In the upgraded pipeline, the Gen-only configuration uses one generator call, while Gen + Ver and Gen + Ver + alg.-filter use two LLM calls, since the deterministic grounding filter is rule-based and does not invoke an LLM. The result supports the narrow conclusion that the upgraded generator-verifier-filter configuration reduces the number of LLM calls from seven to two, while preserving the same downstream planning structure.

**TABLE 3:** LLM-call count comparison. The prior 6-sample final pipeline requires six generator calls and one verifier call. The deterministic grounding filter does not require an additional LLM call.

Pipeline variant	LLM calls
Prior 6-sample ensemble + ver + alg.-filter	7
Upgraded gen-only	1
Upgraded gen + ver	2
Upgraded gen + ver + alg.-filter	2

### 6.2 Pilot live usability evaluation: old GUI vs. new GUI

The live deployment workflow was evaluated by comparing the old GUI with the new GUI. The focus is on operator effort and ease of use during human-in-the-loop planning and plan checking. The study used an exploratory within-subject A/B design in which each participant completed tasks with both interfaces. The interface order was counterbalanced: six participants used the old GUI before the new GUI, and the remaining six followed the reverse order. Each participant completed two timed trials per UI, for a total of four trials. A total of N=12 participants were recruited, with mixed experience using LLM tools and robotics interfaces. The pilot was not designed for comparisons stratified by participant expertise. All participants completed the study individually, with the same moderator and the same instructions. All trials used the same workstation, display setup, network, and model endpoint for both UIs. Before timing, participants received a short standard training session, so the timed runs measured interface use rather than first exposure to the task.

Each interface condition included two timed trials using preassigned scenario-question pairs from the same EV-battery perception-to-plan-acceptance workflow. In each trial, the participant inspected the available detections, formulated or constructed the assigned request, reviewed the generated result, and accepted or rejected the final list.

For each trial, the moderator showed the assigned scenario and question ID. Timing started when the scenario appeared and stopped when the participant did an action on the final list. During timed trials, the moderator could repeat the written instruction or resolve technical faults but did not advise participants on target selection, plan correctness, or interface strategy. The evaluation measured three UI outcomes: time-to-accepted-answer, in seconds; NASA-TLX overall workload, collected once after each test; and UI preference, reported as old GUI or new GUI with a short explanation.

The pilot was designed for descriptive comparison. Completion times, raw NASA-TLX scores, and preference counts are reported descriptively, without claims of statistical significance. Figure 6 shows the completion time for the first and second tasks completed with each interface. Each bar represents 12 participant observations, with each reported attempt corresponding to a different user request. The new GUI reduces completion time relative to the old GUI: the mean time decreases from 46.17s to 27.00 s on the first task and from 36.25s to 23.00 s on the second task.

**FIGURE 6:**
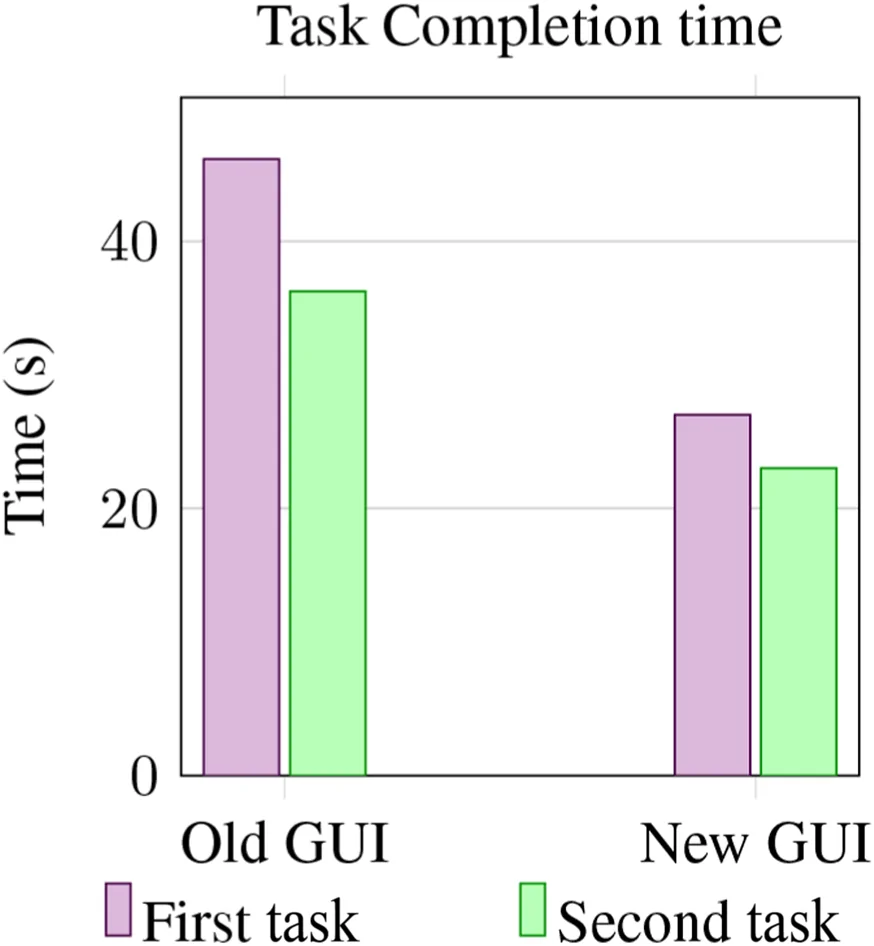
Task completion time in the live GUI study. Bars report the mean completion time across 12 trials for each interface (Old/New), shown separately for the first and second task.

NASA-TLX workload was also lower for the new GUI (UI-A) than for the old GUI (UI-B): mean 14.08 vs. 24.60 and median 10.33 vs. 16.00, out of 100. Table 4 summarizes these results. Finally, all participants reported the new GUI (UI-A) as easier to use than the old GUI (UI-B). Across open-ended responses, the most frequent reasons were reduced typing effort thanks to object buttons, a clearer visual organization with less clutter, and faster object-to-control mapping. Participants also consistently commented on the visual design: the dark background and the more colorful, consistent encodings were perceived as easier to read than the lighter, more visually crowded baseline, and the combination of color cues with numeric IDs helped them distinguish objects and follow instructions.

**TABLE 4:** Raw NASA-TLX overall workload (0–100), retrospective per UI.

Metric	Old GUI (UI-B)	New GUI (UI-A)
Mean	24.60	14.08
Median	16.00	10.33
Participants (count)	12	12

### 6.3 Discussion of results

The offline component study shows that verification and deterministic grounding support different parts of the upgraded planning pipeline. The generator-only configuration already achieves strong full-list correctness, suggesting that the replacement backbone, revised prompts, and structured scene representation support reliable plan formation within the tested benchmark. Adding the verifier improves performance by correcting structural problems, including missing prerequisites, duplicate entries, ordering errors, and formatting drift. The deterministic grounding filter then adds a further gain by rejecting hallucinated or non-matching references. As a result, the final output is both more logically consistent and more tightly tied to the perceived scene.

Evaluating the replacement LLM within the prior pipeline partially separates model replacement from the remaining system changes. Relative to the original prior-pipeline result, mean full-list correctness was 7.20 percentage points higher. The final upgraded configuration provided a further 6.30-point increase with the same replacement LLM, reflecting the remaining pipeline changes as a group. Within the upgraded stack, the verifier and deterministic grounding filter provide the separately measured incremental gains. The results also suggest that, for this constrained benchmark, a capable instruction-following LLM model can be integrated into the existing generator-verifier-filter structure without redesigning the logic. Broader claims about model generalization would require evaluation across multiple LLM families under the same protocol.

The live usability study shows that interface design affects human-in-the-loop interaction. The new GUI reduced the time to an accepted answer on both attempts, lowered NASA-TLX workload, and was preferred by all participants due to improvements in control simplicity and layout. Participants reported that exposing detections, prompts, intermediate LLM outputs, verifier results, and filtered plans made the planning process easier to supervise and increased their comfort with the interface, this supports the value of inspectable interfaces for human-in-the-loop disassembly planning.

### 6.4 Limitations and future work

The results should be interpreted within the operating limits of the current system. The offline benchmark should also be interpreted as a controlled regression benchmark. Its strength is that it uses the same scenes, intents, ground-truth lists, and scoring rules as the prior pipeline, enabling direct comparison. However, accuracy may overestimate performance on unseen battery layouts, object distributions, or operator instructions.

Planning quality is evaluated from structured scene representations, so the work does not yet address general-purpose perception or deeper multimodal scene understanding. Perception errors propagate directly into the structured scene representation. A false negative removes an object, preventing the generator and verifier from including it as a target or prerequisite. A false positive can enter the scene text and pass the deterministic grounding filter because the filter checks consistency with the perceived representation rather than physical truth. Localization or centroid errors can alter inferred stacking, proximity, or blocker relations, while duplicate detections can create redundant instance identifiers or ambiguous operator selections. The verifier and filter constrain LLM outputs relative to the scene text but do not validate the perception representation itself.

The improved performance of the single-model configuration suggests that model capability and instruction alignment can matter more than sampling diversity in this setting. A stronger instruction-following model, combined with structured inputs and strict output rules, reduced the need for ensemble-based candidate exploration in this benchmark, as measured by the number of required model calls. However, this conclusion is tied to the current task and benchmark, where the input is highly structured and the expected answer is a constrained list. More open-ended planning tasks may still benefit from multiple candidates.

The strict grounding filter creates a trade-off between safety and flexibility. It prevents hallucinated outputs and ensures that accepted actions match objects in the structured scene representation. This scene-membership check does not guarantee completeness or precedence. Removing a required line can leave the plan incomplete, so the implementation relies on operator inspection after filtering. At the same time, it may reject otherwise valid plans if the format, object name, or numerical precision differs from the expected representation. Several failure modes remain, including missed precedence relationships, omitted intermediate objects, and valid outputs rejected by strict matching. The GUI evaluation used a within-subject pilot with N=12 . The reported means, medians, and unanimous preference describe the tested participants and tasks rather than population-level effects.

Future evaluation could use equivalence classes of valid plans, so alternative but correct removal orders are not penalized as failures. Improved handling of perception uncertainty would also help the planner reason when object positions or obstruction relationships are unclear. Future work should also improve perception and scene understanding. Segmentation-based methods such as SAM could support more general object detection and broaden the system beyond the current setup. Another direction is a modular multimodal pipeline, where a specialized VLM extracts scene context and semantic information while the LLM focuses on instruction following and plan synthesis. Richer contextual grounding may help the planner operate more reliably in complex scenes and move closer to domain-specific expert behavior.

A further limitation concerns LLM generalization. The upgraded pipeline was evaluated with one main replacement backbone, so the results should not be interpreted as proof of model-agnostic performance. Although the generator-verifier-filter architecture is modular, the reported accuracy may depend on the instruction-following behavior of the selected model. Future work should compare multiple LLM families under the same prompts, decoding settings, benchmark cases, and scoring protocol.

## 7 Conclusion

This paper presented an extended disassembly planning system for intent-driven EV battery disassembly, combining structured RGB-D scene text, a replacement LLM backbone, revised generator and verifier prompts, deterministic grounding, and an inspectable human-in-the-loop interface. On the established 200-scene, 600-intent regression benchmark, full-list correctness increased from 0.824 for the prior 6-sample final pipeline to 0.896 when the replacement LLM was used in the prior pipeline and then to 0.935 with the upgraded generator alone, and to 0.959 when the verifier and deterministic grounding filter were enabled. The 0.824-to-0.896 comparison measures the backbone-replacement difference within the prior pipeline, whereas the 0.896-to-0.959 comparison measures the remaining pipeline changes as a group with the replacement backbone fixed.

The upgraded final configuration also reduced the LLM-call count from seven to two while preserving the downstream generator-verifier-filter structure. In this constrained benchmark, the results suggest that the pipeline can support backbone replacement without relying on ensemble sampling, provided that the input scene text and output rules remain tightly constrained. The experiment evaluates one replacement backbone and therefore does not establish model-agnostic performance across LLM families.

The live usability study with 12 participants provided pilot evidence of improved operator interaction with the redesigned interface. Mean time to accepted answer decreased from 46.17s to 27.00 s on the first task and from 36.25s to 23.00 s on the second task. NASA-TLX workload decreased from 24.60 to 14.08, and all participants preferred the new interface. Overall, the study suggests that LLM-based disassembly planning can be made more reliable and inspectable when model outputs are constrained, checked against the perceived scene, and exposed to the operator before execution.

## Data Availability

Data are available from the corresponding author upon reasonable request.

## Footnotes

1 Under full-list evaluation, enabling both verification and filtering yields the best performance (95.9%).

2 In live deployment, 3D point-cloud reconstruction supports operator inspection and fused depth contributes to centroid estimation. The offline benchmark bypasses both functions because it uses precomputed structured scene text.
