# Supplementary material for: Interactive and inspectable LLM planning for human-in-the-loop EV battery disassembly
Source: Front Robot AI. 2026 Oct 12;13:1916454. doi: 10.3389/frobt.2026.1916454 (PMC13696855; doi:10.3389/frobt.2026.1916454)
Supplement: Supplementary file 1 [file Supplementaryfile1.pdf]

Supplementary Appendix

Interactive and Inspectable LLM Planning for
Human-in-the-Loop EV Battery Disassembly

Table A5 summarizes the core notation used in the mathematical formulation, while Table A6 reports the implementation parameters of the deployed pipeline. Table A7 reports the LLM/API configuration used for the upgraded generator-verifier pipeline. The seeds document the request configuration, but inference through a remote distributed provider does not guarantee bit-level determinism.

Table A5: Core notation used in the mathematical formulation.

Symbol	Meaning
x_t	RGB-D sensor state at time t.
σ_t	Structured scene text in Cartesian XYZ coordinates.
D_t	Rewritten scene text in LUZD form, $D_t = T(\sigma_t)$.
U_t	User-side instruction payload (scene + operator intent).
p_t^{gen}	Generator message payload (system + user roles).
g_t, v_t	Generator and verifier raw outputs, respectively.
$\tilde{r}_t$	Cleaned generator list used as verifier input context.
$z_t^{\text{raw}}, z_t^{\text{da3}}, z_t^{\text{fused}}$	Raw, DA3, and fused depth maps.
$\chi_t^{(n)}$	Deterministic acceptance bit for candidate line n.
A_t	Final accepted output list after deterministic filtering/deduplication.

Table A6: Key implementation parameters used in the deployed pipeline.

Parameter	Value	Role
W	20	Temporal depth window size for robust raw-depth aggregation.
f_{sample}	0.2	Pixel sample fraction for robust scale-shift fitting.
k_{MAD}	3.0	MAD inlier threshold multiplier in robust fitting.
σ_{mad}	0.01	Decay scale (m) for MAD-based raw-depth reliability weighting.
σ_{edge}	0.02	Edge gate scale (m/px) to prevent residual bleeding across depth boundaries.
k_{blur}	31	Kernel size for smooth residual-field correction.
δ	0.5	Residual correction clipping limit (m).
k_{med}	5	Median-filter kernel for spike suppression.
τ_{spike}	0.22	Spike replacement threshold in fused depth (m).
r_{dedupe}	0.01	3D radius threshold for detection deduplication (m).

Table A7: LLM/API configuration for the upgraded generator-verifier pipeline.

**Parameter**	**Value**
Provider	Featherless.ai
API type	OpenAI-compatible chat-completions API
Endpoint	`https://api.featherless.ai/v1`
Model identifier	`moonshotai/Kimi-K2-Instruct-0905`
Temperature	0.2
Top-p	0.4
Top-k	-1
Min-p	0.06
Repetition penalty	1.04
Maximum output tokens	4096
Context length	32768
Generator seed	21
Verifier seed	2025
